# Efficacy and safety of recombinant human follicle-stimulating hormone in patients undergoing *in vitro* fertilization-embryo transfer

**DOI:** 10.18632/aging.102919

**Published:** 2020-03-25

**Authors:** Linli Hu, Songying Zhang, Song Quan, Jieqiang Lv, Weiping Qian, Yuanhua Huang, Weiying Lu, Yingpu Sun

**Affiliations:** 1 The First Affiliated Hospital of Zhengzhou University, Reproductive Medicine, Zhengzhou, China; 2 Sir Run Run Shaw Hospital of Zhejiang University, Reproductive Medicine, Hangzhou, China; 3 Southern Medical University, Reproductive Medicine, Guangzhou, China; 4 Second Affiliated Hospital of Wenzhou Medical University, Reproductive Medicine, Wenzhou, China; 5 Peking University Shenzhen Hospital, Reproductive Medicine, Shenzhen, China; 6 Hainan Medical College, Reproductive Medicine, Haikou, China

**Keywords:** recombinant human FSH (r-hFSH), ovarian stimulation, assisted reproductive technology (ART), oocytes retrieved, IVF-ET

## Abstract

To compare the ovarian responses after administration of two recombinant follicle-stimulating hormone (r-FSH) preparations under gonadotropin-releasing hormone (GnRH) analogue downregulation, we conducted a phase 3, randomized, multicenter, assessor-blind, active-controlled, parallel group study. The primary outcome was the number of oocytes retrieved. The secondary outcomes included total dose and duration of r-FSH administered, oocyte quality, blood estradiol levels, follicular development, fertilization rates, implantation rates, and pregnancy rates (biochemical, clinical, and ongoing). A total of 451 patients with infertility were randomized to receive either Follitrope™ Prefilled Syringe or Gonal-F^®^ Pen for ovarian stimulation. The mean number of oocytes retrieved was 14.9 in the Follitrope^TM^ Prefilled Syringe group, and 12.8 in the Gonal-F^®^ Pen group. The 95% confidence interval in the oocyte number difference between the groups was [–0.1, 4.2], demonstrating that Follitrope^TM^ Prefilled Syringe was not inferior to Gonal-F^®^ Pen. The clinical pregnancy rates (Follitrope^TM^ Prefilled Syringe vs. Gonal-F^®^ Pen: 55.4% vs. 51.9%) and ongoing pregnancy rates (44.1% vs. 43.0%) were similar between the groups. No clinically significant adverse events were observed in either group. In summary, our study indicates that Follitrope^TM^ Prefilled Syringe is safe and efficacious for ovarian stimulation.

## INTRODUCTION

Infertility is defined as a failure to achieve a clinical pregnancy after 12 months or more of regular unprotected sexual intercourse [1]. Approximately 48.5 million couples worldwide experience infertility [2]; they may try to achieve pregnancy with assisted reproductive technology (ART). To increase the probability of obtaining embryos with implantation potential, a multifollicular development by ovarian stimulation is required. Since the first *in vitro* fertilization (IVF) birth in 1978 [3], the IVF/intracytoplasmic sperm injection (ICSI) techniques with controlled ovarian stimulation (COS) have become a common practice for infertile couples. The ovarian stimulation can be achieved with pituitary desensitization followed by administration of gonadotropins. Alternatively, it can be induced by gonadotropin administration after suppression of premature luteinizing hormone (LH) surge with gonadotropin-releasing hormone (GnRH) agonist. Gonadotropins then stimulate ovaries to produce multiple dominant follicles that yield multiple oocytes for fertilization.

The first gonadotropin medication introduced to clinical practice was the human menopausal gonadotropin (hMG), which was purified from urine of menopausal women [4]. To improve the safety and convenience of the administration of human gonadotropins, highly purified urinary follicle-stimulating hormone (HP-uFSH) and hMG (HP-hMG) were developed and used for more than four decades [5–8]. Although their purity increased compared to the initial preparations, they were still contaminated with urine proteins that might cause side effects, such as allergic reactions. Moreover, the dependence on urine collection represented another problem. Development of the commercially produced recombinant FSH (r-FSH) by using recombinant DNA technology produced highly purified and effective FSH preparations, with batch-to-batch consistency for the treatment of infertility [8–10].

The main objective of this study was to compare the ovarian responses in terms of the number of oocytes retrieved after administration of two r-FSH preparations, Follitrope ^TM^ Prefilled Syringe and Gonal-F^®^ Pen with GnRH analogue downregulation. Follitrope^TM^ Prefilled Syringe (LG Chem, Ltd., South Korea) is a r-FSH that consists of follitropin alfa and beta subunits. Its efficacy and safety have already been confirmed in previous phase 3 studies (unpublished data); it has been marketed in more than 13 countries since 2006.

Since earlier studies have indicated that the adjunctive use of GnRH agonist improves the outcomes compared with conventional gonadotropin therapy [11–14], co-treatment of GnRH agonist with gonadotropin has been the mainstay of the COS regimen in IVF practice, especially in younger patients. In this study, we used the traditional GnRH agonist long protocol that has been commonly used for pituitary desensitization prior to administration of gonadotropins [15]. Since the ovarian responses to ovarian stimulation depend on age [16], patients in this study were stratified according to their age. In addition to the number of retrieved oocytes, other efficacy parameters including ongoing clinical pregnancy rates and safety outcomes were compared between the two r-FSH preparations.

## RESULTS

### Patients

**Figure 1:**
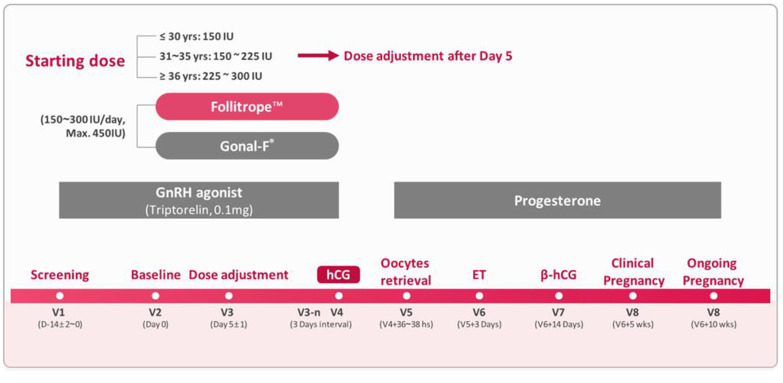
Overview of the study

**Figure 2:**
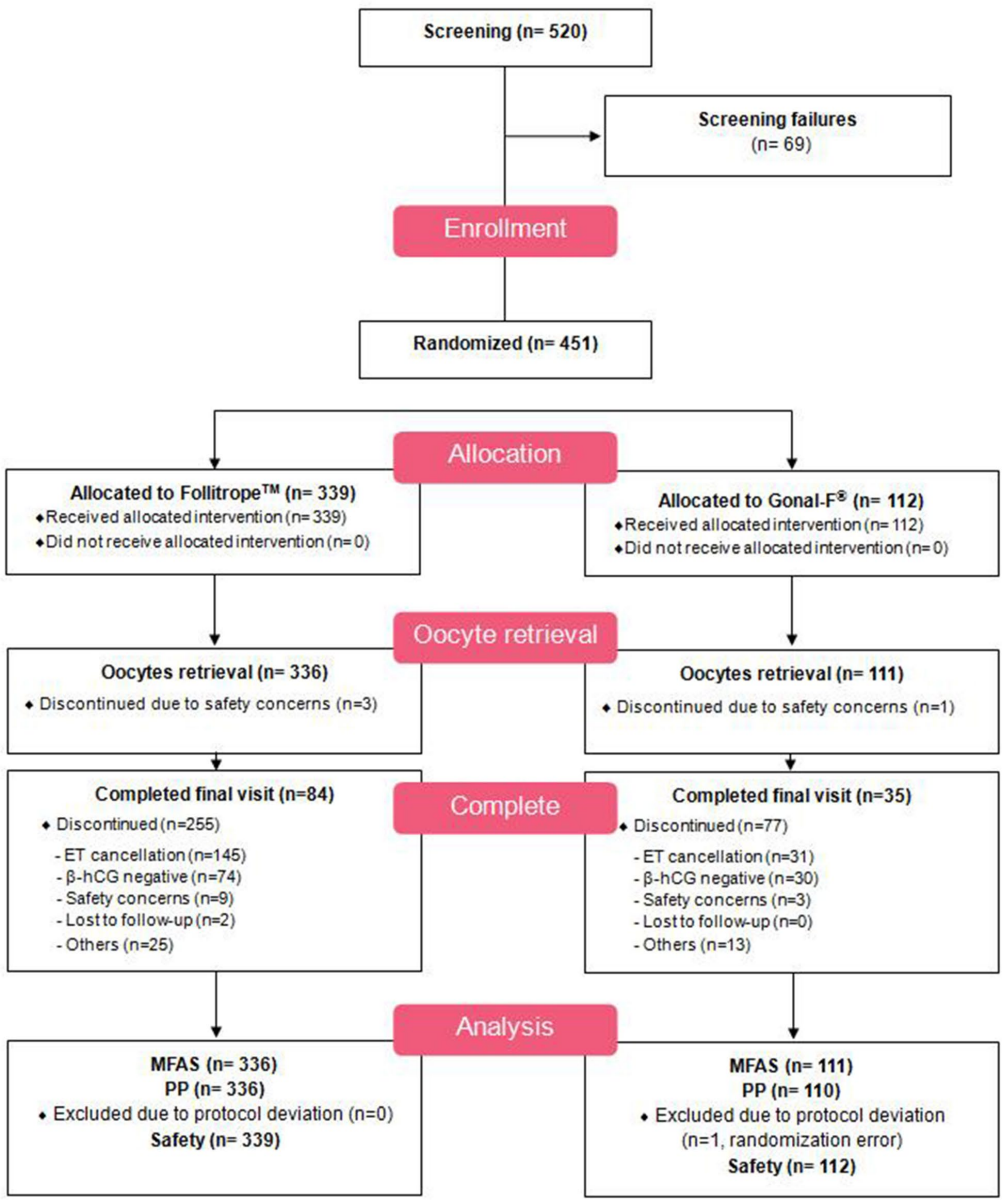
Patient disposition.

From 520 screened patients, 69 patients were excluded; the most common reason for the exclusion was a lack of pituitary suppression. A total of 451 patients were randomized for the use of r-FSH treatment, and 447 patients completed the oocyte retrieval. These patients were included in the modified full analysis set (MFAS). The main reason for discontinuation before oocyte retrieval was a hyper-response. From the 447 MFAS patients, 446 patients were included in the per-protocol (PP) set, which compared the primary efficacy; 1 patient was excluded due to a randomization error. Figure 1 illustrates the overall study design; Figure 2 presents the disposition of patients.

Demographics and baseline characteristics were comparable between the groups (Table 1). Patients’ mean (±SD) age was 29.4 (± 3.8) years, and mean (±SD) BMI was 21.4 (± 2.7) kg/m^2^. The most common cause of infertility was a tubal factor (42.7%); majority (96.6%) of the patients did not undergo a COS treatment before. The mean (±SD) exposure to the GnRH agonist therapy was 14.3(±1.50) days and 14.4 (± 1.23) days in the Follitrope group and Gonal-F group, respectively. Among all patients, the proportions of patients with IVF or ICSI were 52.0% and 41.0%, respectively. Both IVF and ICSI were performed in 7.0% of the patients. Distribution of the patients according to fertilization procedure was similar between the groups (*p* = 0.242).

**Table 1:** Baseline characteristics (MFAS population).

	**Follitrope^TM^ PFS (N = 336)**	**Gonal-F^®^ Pen (N = 111)**	***p*-value**
Age, years^a^	29.4 ± 3.9	29.3 ± 3.6	0.814
Age, n (%)			
20-30 yrs	198 (59)	73 (66)	0.376
31-35 yrs	118 (35)	31 (28)	
36-39 yrs	20 (6)	7 (6)	
BMI, kg/m^2a^	21.4 ± 2.7	21.3 ± 2.6	0.881
Duration of infertility, month^a^	3.9 ± 2.5	3.9 ± 3.1	0.805
Main reason for infertility, n (%)			
Tubal factor	149 (44.3)	42 (37.8)	0.250
Male factor	105 (31.3)	46 (41.4)	
Unexplained	58 (17.3)	15 (13.5)	
Combined	24 (7.1)	8 (7.2)	
Fertilization procedure, n (%)			
IVF	182 (54.2)	50 (45.5)	0.242
ICSI	130 (38.7)	53 (48.2)	
IVF and ICSI	24 (7.1)	7 (6.4)	
Basal FSH level, mIU/ml^a^	3.0 ± 0.9	3.1 ± 1.0	0.827
Basal E_2_ level, pg/mL^a^	21.4 ± 12.4	20.4 ± 10.6	0.498

^a^Values are mean±SD.

### Efficacy

#### Primary outcome

The least square mean (±SD) number of oocytes retrieved was 14.9 (± 0.5; median [range]: 14 [1 to 41]) in the Follitrope group, and 12.8 (± 0.9; median [range]: 13 [3 to 33]) in the Gonal-F group, showing a treatment difference of 2.1 oocytes. The 95% confidence interval (CI) of the oocyte difference between the groups was [–0.1, 4.2]. As the lower limit of the 95% CI was >–3, these data indicated that Follitrope was not inferior to Gonal-F.

A subgroup analysis by age indicated that the results were consistent across the subgroups; the mean (±SD) number of oocytes retrieved were 16.3 (± 7.8) versus 15.4 (± 6.8) (Follitrope vs. Gonal-F group) in the age group of 20-30 years; 14.2 (± 6.8) versus 11.7 (± 4.2) in the age group of 31-35 years; and 12.5 (± 7.0) versus 7.4 (± 3.2) in the age group of 36-39 years. The lower limits of the 95% CIs for the treatment difference were >–3 in all subgroups. Treatment differences in the mean number of oocytes retrieved were 1.0 (95% CI: [–1.1, 3.0]), 2.5 (95% CI: [0.5, 4.5]), and 5.1 [(95% CI: [−0.7, 10.8]) in the 20-30, 31-35, and 36-39 years age groups, respectively (Supplementary Table 1).

#### Secondary outcomes

The results are presented in Table 2. Although the total dose of r-FSH used for ovarian stimulation in each group was comparable (1945.3 IU vs. 2020.2 IU, *p* = 0.271), the treatment duration of the Follitrope group was significantly shorter than in the Gonal-F group (10.7 days vs. 11.1 days; *p* = 0.027). In subgroup analysis, the total r-FSH doses decreased proportionally with the patients’ age.

**Table 2:** Efficacy outcomes (PP population).

	**Follitrope^TM^ PFS (N = 336)**	**Gonal-F^®^ Pen (N = 110)**	**p-value**
Total injected dose of r-FSH, IU^a^	1945.3 ± 635.7	2020.2 ± 562.7	0.271
Duration of treatment, days^a^	10.7 ± 1.6	11.1 ± 1.4	0.027
No. of follicles with diameter of ≥14 mm on hCG day	10.2 ± 4.0	10.1 ± 4.4	0.750
E_2_ concentration on hCG day, pg/mL	4460.0 ± 2564.1	4051.9 ± 2583.5	0.167
No. of oocytes retrieved^a^	14.9 ± 0.5	12.8 ± 0.9	0.022
Metaphase II oocytes rate, %^a,b^	85.3 ±14.0	85.0 ± 15.8	0.895
Fertilization rate, %	70.6	74.6	0.079
No. of embryos transferred^a^	2.0 ± 0.3	2.0 ± 0.4	0.731
Embryo implantation rate, %	39.8 (144/362)	35.5 (55/155)	0.376
Biochemical pregnancy rate, %	4.3 (8/186)	10.1 (8/79)	0.110
Clinical pregnancy rate, %	55.4 (103/186)	51.9 (41/79)	0.168
Ongoing pregnancy rate, %	44.1 (82/186)	43.0 (34/79)	0.758

^a^ Values are mean±SD

^b^ICSI patients only

Follicular development and hormonal characteristics on the day of hCG administration were similar between the groups. The total number of preovulatory follicles with a diameter of ≥14 mm was 10.2 ± 4.0 in the Follitrope group, and 10.1 ± 4.4 in the Gonal-F group (*p* = 0.75). Serum estradiol concentration rose from 21.4 ± 12.4 pg/mL to 4460.0 ± 2564.1 pg/mL in the Follitrope group, and from 20.4 ± 10.6 pg/mL to 4051.9 ± 2583.5 pg/mL in the Gonal-F group (*p* = 0.1669).

With respect to the morphological markers of oocyte quality, the percentages of the metaphase II (MII) oocytes in ICSI patients were 85.3% and 85.0% in Follitrope and Gonal-F groups, respectively (*p* = 0.895). The overall fertilization rate in the Follitrope group was 70.6%, which was comparable to the 74.6% rate in the Gonal-F group (*p* = 0.079). Regarding embryo transfer (ET), more attempts were made in the Gonal-F group than in the Follitrope group. In the Follitrope group, 186 patients (55.4%) underwent ET in contrast to 79 patients (71.8%) in the Gonal-F group; thus, the cancellation rate was 44.3% and 28.2% in the Follitrope and Gonal-F groups, respectively (*p* = 0.0028). The number of transferred embryos in each group was similar (2.0 ± 0.3 in the Follitrope group vs. 2.0 ± 0.4 in the Gonal-F group, *p* = 0.731). Most patients (91.3%) received 2.0 embryos per transfer. After ET, the patients were observed for at least 10 weeks during pregnancy. The percentage of embryos that were successfully implanted in patients showing gestational sacs (i.e., implantation rate) was 39.8% in the Follitrope group and 35.3% in the Gonal-F group (*p* = 0.376).

No significant differences were observed in the pregnancy rates between the Follitrope and Gonal-F groups. A patient was regarded as biochemically pregnant when the serum β-hCG test at 14 days after ET was positive without observation of gestational sac at 5 weeks after ET. The biochemical pregnancy rate was 4.3% in the Follitrope group compared with 10.1% in the Gonal-F group (*p* = 0.110). Clinical pregnancy was confirmed by the presence of a gestational sac using ultrasound at 5 weeks after ET; the rates were 55.4% in the Follitrope group, and 51.9% in the Gonal-F group (*p* = 0.168). The ongoing pregnancy rate, defined as the percentage of pregnancies with fetal heart rate maintained for at least 10 weeks after ET, was 44.1% and 43.0% in the Follitrope and Gonal-F groups, respectively (*p* = 0.758).

Pregnancy rates in each age subgroup were similar between the treatment groups; as expected, the rates decreased in the 36-39 years age group. The clinical pregnancy rates in each age subgroup were 55.1% (20-30 years), 60.8% (31-35 years), and 28.6% (36-39 years) in the Follitrope group, and 58.7% (20-30 years), 51.9% (31-35 years), and 0% (36-39 years) in the Gonal-F group (Supplementary Table 1).

#### Safety

The overall incidence rate of adverse events (AEs) was comparable between the groups (Table 3). Most AEs were mild to moderate in their severity. The most common drug-related AE was the ovarian hyperstimulation syndrome (OHSS). The proportion of patients with OHSS was 27.4% in the Follitrope group, and 21.4% in the Gonal-F group. Severe OHSS occurred in 1.2% of patients in the Follitrope group, and 4.5% in the Gonal-F group. Hyper-response to COS was the most common reason for the cancellation of ET. Gastrointestinal disorders, including abdominal distention and nausea, were the most commonly reported AEs excluding OHSS.

**Table 3:** Summary of TEAEs (Safety population).

	**Follitrope^TM^ PFS (N = 339)**	**Gonal-F^®^ Pen (N = 112)**	***p*-value**
No. of patients with any AEs, n (%)	204 (60.2)	61 (54.5)	0.3194
No. of patients with most frequently reported AEs, n (%)			-
OHSS	93 (27.4)	24 (21.4)	
Abdominal distension	33 (9.7)	14 (12.5)	
Vaginal infection	18 (5.3)	7 (6.3)	
Nausea	9 (2.7)	6 (5.4)	
No. of patients with SAEs, n (%)	11 (3.2)	8 (7.1)	0.1001
No. of patients with solicited local reactions, n (%)			-
Any local reactions	27 (8.0)	6 (5.4)	
Irritation	1 (0.3)	1 (0.9)	
Haemorrhage	1 (0.3)	0 (0)	
Warmth	5 (1.5)	0 (0)	
Erythema	2 (0.6)	0 (0)	
Bruising	2 (0.6)	1 (0.9)	
Pain	5 (1.5)	0 (0)	
Induration	12 (3.5)	4 (3.6)	
Mass	2 (0.6)	0 (0)	
Swelling	2 (0.6)	1 (0.9)	

AE= Adverse event; SAE= Serious adverse event; OHSS= Ovarian hyperstimulation syndrome.

The incidence of local reactions was similar between the groups; induration was reported in about 3.5% of patients in each group, followed by pain and fever. No clinically significant changes in vital signs, laboratory results, or physical examinations were found. No patients showed any newly developed anti-FSH antibodies after the treatment.

## DISCUSSION

Our results show that Follitrope is non-inferior to Gonal-F with regard to the number of oocytes retrieved, which is the recommended primary endpoint for comparative clinical trials of r-FSH [17]. Patients’ characteristics and outcomes, including the number of oocytes and the ongoing pregnancy rates were within the range reported in previous studies of Gonal-F [18–23].

The ultimate objective of ART is to achieve a healthy live birth. In this context, it has been suggested that the main outcome measure for reproductive medicine should be the live birth rates or ongoing clinical pregnancy rates [24–30]. However, in the case of multicenter trials to compare the therapeutic effects of different medications, it is important to consider that there could be unquantifiable biases and confounding factors that originate from patients (e.g., sperm quality, endometrial thickness) or procedures following oocyte collection (e.g., fertilization, embryo culture). Therefore, we decided to use the number of oocytes retrieved as the primary outcome measure for this study, considering that the purpose of ovarian stimulation with r-FSH preparations is basically to produce multiple oocytes to facilitate IVF. The success of ART depends on the collection of a sufficient number of oocytes that could develop into good-quality embryos, without AEs such as OHSS. Indeed, several prior studies have reported that the live birth rates are closely associated with the number of oocytes obtained; the optimal number of retrieved oocytes to maximize the live birth rate without OHSS risk ranges from 10 to 15 [31–33].

In our study, the pregnancy rates also correlated with the number of retrieved oocytes. Treatment duration and total dose of r-FSH used were comparable between the two groups; even though the difference in the treatment duration between the groups was statistically significant, it was not clinically meaningful (10.7 vs. 11.1 days, *p* = 0.027). About 70% of retrieved oocytes succeeded in achieving fertilization, with no significant differences between treatment groups. Finally, the pregnancy rates of both treatment groups did not differ from the outcomes reported in annual reports of the Society for Assisted Reproductive Technology and Centers for Disease Control and Prevention [34, 35].

Our subgroup analysis indicates that Follitrope might be more favorable for older patients. Although there were no statistically significant differences because of the small sample size, more oocytes were retrieved in the Follitrope group than in the Gonal-F group in all age subgroups. The number of retrieved oocytes was within the range of the optimum number of oocytes for a good IVF prognosis (i.e., 10-15 oocytes), and therefore resulted in satisfactory pregnancy rates. However, a tendency towards worse outcomes was observed in older patients in both treatment groups. In addition, Follitrope was found to have a favorable safety profile compared with Gonal-F. No unexpected safety concerns were identified in this study, but the OHSS incidence rate was somewhat higher than the rate reported in other studies, regardless of the treatment group [18–21, 23, 36–40].

Several factors affect the prognosis of IVF/ICSI. One of the critical factors is the patient’s ovarian response to treatment [16, 41]; this response is associated with the patient’s age and ovarian markers, such as antral follicle count (AFC) or serum anti-Mullerian hormone (AMH). In this aspect, much attention has been focused on the individualization of COS as an alternative therapeutic strategy to raise cost-effectiveness and reduce the risk of OHSS [42, 43]. Individualized COS is initiated with a tailored dose of gonadotropin based on the patient’s ovarian potential estimated by the above factors. The starting doses of r-FSH used in this study were stratified according to patient’s age instead of using a conventional initial r-FSH dose. As a result, there was a trend toward a reduced use of r-FSH in younger patients despite better outcomes (Supplementary Table 1).

One potential limitation of this study is that the baseline AMH or AFC levels were not considered as a randomization factor. However, this potential bias may not represent a significant concern when generalizing the study findings, considering that other patients’ characteristics were comparable between the groups. Nevertheless, a plausible imbalance in AMH or AFC levels between the groups might have influenced the treatment difference in the cycle cancellation rate, which mainly resulted from ovarian hyper-response. In fact, Rettenbacher et al. [21] reported that a high AMH level was related to the incidence of OHSS in their phase 3 trial of r-FSH, which targeted a patient population similar to our study. In addition, the high OHSS incidence rate might have been caused by the fact that the initial doses were based only on patient’s age, even though they were adjusted 5 days later. Actually, OHSS was reported in <10% of patients (data not published) in previous randomized trials of Follitrope, in which the conventional r-FSH starting dose (i.e., 150 IU/day) was used. Another limitation of our study is that the live birth rate, which is the most relevant clinical endpoint for infertility treatment, is not available. Thus, further studies are needed to predict prognosis, including the live birth rate, in patients receiving the Follitrope^TM^ Prefilled Syringe treatment. However, despite the above limitations, our study shows that both treatments have comparable results as well as acceptable pregnancy rates.

In conclusion, our study demonstrates that the Follitrope^TM^ Prefilled Syringe treatment is well tolerated and not inferior to Gonal-F^®^ Pen treatment, indicating that it will be beneficial to IVF-ET patients. Therefore, Follitrope is another treatment option for stimulation of follicular development in infertile women undergoing ART.

## MATERIALS AND METHODS

### Study design

This phase 3, randomized, multicenter, assessor-blind, active-controlled, parallel-group study was conducted in 6 IVF centers in China (The First Affiliated Hospital of Zhengzhou University, Sir Run Run Shaw Hospital of Zhejiang University, Southern Medical University, Second Affiliated Hospital of Wenzhou Medical University, Peking University Shenzhen Hospital, Hainan Medical College, Reproductive Medicine). Figure 1 illustrates an overview of the study treatments and procedures. Written informed consent was obtained from all patients. After screening for eligibility, the conventional long GnRH agonist regimen was initiated. The GnRH agonist triptorelin (Decapeptyl; DeBiopharm, Germany) was administered daily for 14 days (± 2 days) according to local practice, and then eligible patients were randomized to a test group (Follitrope ^TM^ Prefilled Syringe) or a control group (Gonal-F^®^ Pen; Merck Serono, Italy) in a 3:1 ratio. Randomization was stratified by age (20–30, 31–35, and 36–39 years). Random numbers were generated by an independent statistician using SAS 9.2 software (SAS Institute, USA), and patients were randomized by opening sealed, sequentially numbered envelopes. The r-FSH preparations were administered via subcutaneous injections concomitantly with the GnRH agonist. This study regimen was maintained until the day of ovulation induction. Thereafter, oocyte retrieval followed by IVF/ICSI, and ET were conducted. The patients were followed up for at least 10 weeks after ET to confirm their ongoing pregnancy.

The protocol was approved by the Institutional Review Board of each IVF center and the China Food and Drug Administration (CFDA) for phase 3 trials. This study was conducted in accordance with the Declaration of Helsinki and good clinical practice guidelines.

### Study population

The study drugs were indicated for COS in patients undergoing IVF-ET based on the following factors: tubal factors, unexplained infertility, male infertility, and combined factors. Chinese women aged 20 to 39 years with a normal menstrual cycle (25 to 35 days) were screened for eligibility. Patients with an abnormal basal level of serum FSH, LH, estradiol, or progesterone; body mass index (BMI) >30 kg/m^2^; a history of OHSS; previous poor ovarian responses; or a failure of IVF cycle more than three times in the past were excluded. Finally, patients who achieved downregulation of pituitary after initial GnRH agonist therapy were enrolled in this study. Additional inclusion and exclusion criteria are provided in the Supplementary File.

### Treatment

The GnRH agonist (0.1 mg of triptorelin, subcutaneous injection once daily) was started in the mid-luteal phase of the previous menstrual cycle. After randomization, the dose of GnRH agonist was reduced to 0.05 mg and maintained until the day of ovulation induction. Pituitary downregulation was considered to be achieved when the median antral follicle size measured by vaginal ultrasound was <10 mm. The r-FSH preparations were administered only to the patients who achieved pituitary downregulation. Patients could not be blinded because of the difference in appearance between the two FSH preparations. Physicians and evaluators (including embryologists and central laboratory personnel) were blinded until the end of this study.

The initial dose of FSH was determined according to the patient’s age: 150 IU/day in women aged 20-30 years, 150 to 225 IU/day in women aged 31-35 years, and 225-300 IU/day in women aged >35 years. The dose could be adjusted based on the patient’s response as early as from day 5. An incremental adjustment in dose from 75 IU was permitted with a maximum daily dose of 450 IU at an interval of 3 days. When the patient met one of the following criteria, treatment was discontinued, and an intramuscular injection of human chorionic gonadotropin (hCG; 5000-10,000 IU) was administered to induce ovulation: 1) one or more follicles with a diameter of ≥18 mm or 2) three or more follicles with a diameter of ≥16 mm. Oocytes were retrieved 36 to 38 hours after hCG injection. Just after the oocyte retrieval, retrieved oocytes were fertilized via conventional IVF or ICSI. Fertilized embryos were cultured in the medium for 3 days. After that, the embryos were graded for morphology according to the grading system introduced by Veeck [44], and qualified embryos (Grade I–Grade II; embryos with <20% fragmentation) were transferred to the patient. Only one cycle of ET per patient was allowed in this study. The luteal phase was supported by progesterone administered intramuscularly once a day (60 mg/day) for 10 weeks after ET.

### Outcome measures

The primary endpoint of this study was the number of oocytes retrieved. The oocytes were retrieved from each patient via ultrasound-guided transvaginal needle aspiration. Secondary efficacy endpoints included total cumulative dose of r-hFSH, duration of stimulation, blood estradiol levels, total numbers of follicles with a diameter of ≥14 mm on hCG day, oocyte maturation rates (percentage of metaphase II oocytes, ICSI only), fertilization rates, embryo implantation rates, number of embryos transferred, and three distinct pregnancy rates: biochemical, clinical, and ongoing pregnancy.

Follicular diameter was measured via vaginal ultrasound. Fertilization rate was calculated as the percentage of the number of 2PN oocytes divided by the number of oocytes used for fertilization (IVF) or number of microinjection of metaphase II oocytes (ICSI). Pregnancy rates were defined as the number of pregnancies per 100 initiated ET cycles [1]. Vital signs, laboratory test results, and AEs including OHSS were monitored as safety endpoints. Local reactions at the injection sites were also evaluated. The level of anti-FSH antibodies was monitored using validated electrochemiluminescence immunoassay (Meso Scale Discovery Sector Imager 6000; Meso Scale Diagnostics, LLC., USA) to evaluate the immunogenicity of the r-hFSH preparations at baseline and at the end of the study.

### Sample size calculation and statistical analyses

The aim of the study was to compare the Follitrope^TM^ Prefilled Syringe with the Gonal-F^®^ Pen in terms of the number of oocytes retrieved. Based on the previous equivalence trials of Gonal-F [15–17], the sample size was determined to detect a difference of at least three in the mean number of oocytes retrieved between groups with a power of 80% and significance level of 5%. Assuming a 20% dropout rate, 464 patients were randomized in a 3:1 ratio.

For all efficacy outcomes, summary statistics (mean, SD, median, minimum, maximum, and quartile) for each group were presented, and the 95% CI of the treatment difference between groups was assessed. As this study was designed as non-inferiority trial, the lower limit of the 95% CI was compared with a pre-specified non-inferiority margin (i.e., –3 oocytes) in the primary analysis. To compare the primary efficacy endpoints, an analysis of variance model was used with treatment group, site, and treatment as fixed categorical effects.

To compare other efficacy outcomes between the two groups, statistical tests were performed using Fisher’s exact test or Student *t*-test depending on the characteristic of the variable. Safety analysis, modified full analysis, and PP analysis were conducted. Subgroup analysis according to patient age was also performed for all efficacy outcomes using *t*-test. The patients were grouped into three subgroups according to their age: 20-30 years, 31-35 years, and 36-39 years. Regarding the safety analysis, local reaction profiles, changes in vital signs, and laboratory test results were compared in addition to the incidence of the AEs.

## Supplementary Material

Supplementary File

Supplementary Table 1
