# Correction for: Efficacy and safety of recombinant human follicle-stimulating hormone in patients undergoing *in vitro* fertilization-embryo transfer

**DOI:** 10.18632/aging.204490

**Published:** 2023-06-29

**Authors:** Linli Hu, Songying Zhang, Song Quan, Jieqiang Lv, Weiping Qian, Yuanhua Huang, Weiying Lu, Yingpu Sun

**Affiliations:** 1 The First Affiliated Hospital of Zhengzhou University, Reproductive Medicine, Zhengzhou, China; 2 Sir Run Run Shaw Hospital of Zhejiang University, Reproductive Medicine, Hangzhou, China; 3 Southern Medical University, Reproductive Medicine, Guangzhou, China; 4 Second Affiliated Hospital of Wenzhou Medical University, Reproductive Medicine, Wenzhou, China; 5 Peking University Shenzhen Hospital, Reproductive Medicine, Shenzhen, China; 6 Hainan Medical College, Reproductive Medicine, Haikou, China

**Keywords:** recombinant human FSH (r-hFSH), ovarian stimulation, assisted reproductive technology (ART), oocytes retrieved, IVF-ET

**This article has been corrected:** The authors found several small errors and miscalculations, which were corrected, making text consistent with the Supplemental Clinical Study Final Report:

## Table 1. Baseline characteristics (MFAS population).

**7^th^ row: **The unit “month” for the “Duration of infertility” was corrected to “year”, making it consistent with the Supplemental Clinical Study Final Report.

**13th row: **The *p*-value (0.242) for “Fertilization procedure, n (%)” was corrected to “0.212”. In addition, a footnote “b” (The total number of evaluable patients was 110 in the Gonal-F® Pen group)” was added.

## RESULTS, Patients

The p-value in the last sentence in the **Patients** section was changed from *p* = 0.242 to *p*= 0.212.

**Corrected sentence:** “The distribution of the patients according to fertilization procedure was similar between the groups (*p*= 0.212).” This is now consistent with value in the corrected **Table 1**.

## RESULTS, Efficacy – Primary outcome

In the first sentence of the “*Primary outcome*” section, the abbreviation "±SD” was corrected to "±SE”, and the number 13 was corrected to 13.5. This SE value was estimated from an ad hoc analysis for this manuscript; the analysis source (SAS output) is available. The number 13.5 was based on a result from the Clinical Study Final Report (Table 15, available).

**Corrected sentence:** “The least square mean (±SE) number of oocytes retrieved was 14.9 (± 0.5; median [range]: 14 [1 to 41]) in the Follitrope group and 12.8 (± 0.9; median [range]: 13.5 [3 to 33]) in the Gonal-F group, showing a treatment difference of 2.1 oocytes.”

## RESULTS, Efficacy – **Secondary outcome**

A subtraction error was corrected in the following sentence, which changed 44.3% to 44.6%.

**Corrected sentence:** “In the Follitrope group, 186 patients (55.4%) underwent ET in contrast to 79 patients (71.8%) in the Gonal-F group; thus, the cancellation rate was 44.6% and 28.2% in the Follitrope and Gonal-F groups, respectively (p = 0.0028).”

A numerical error was also corrected in the following sentence, which changed 35.3% to 35.5%.

**Corrected sentence:** “The percentage of embryos that were successfully implanted in patients showing gestational sacs (i.e., implantation rate) was 39.8% in the Follitrope group and 35.5% in the Gonal-F group (p = 0.376).” This correction makes the stated numbers consistent with **Table 2**.

## Table 2. Efficacy outcomes (PP population).

**3rd and 4th rows: **For “No. of follicles with diameter of ≥14 mm on hGC day” and “E2 concentration on hGC day, pg/mL), a footnote “a” was added based on the final result from the Clinical Study Final Report (Tables 26 and 36).

**5th row: **For “No. of oocytes retrieved” the footnote “c” (Values are mean±SE)” was added, as requested above for the “*Primary outcome*” section in the RESULTS. In addition, the *p*-value was corrected from “0.022” to “0.057” based on the final result from the Clinical Study Final Report (Table 16).

Corrected **Tables 1** and **2** are presented below.

**Table 1:** Baseline characteristics (MFAS population).

		**Follitrope^TM^ PFS** **(N = 336)**		**Gonal-F^®^ Pen** **(N = 111)**		***p*-value**	
Age, years^a^	29.4 ± 3.9		29.3 ± 3.6		0.814	
Age, n (%)					0.376	
20-30 yrs	198 (59)		73 (66)			
31-35 yrs	118 (35)		31 (28)			
36-39 yrs	20 (6)		7 (6)			
BMI, kg/m^2a^	21.4 ± 2.7		21.3 ± 2.6		0.881	
Duration of infertility, year^a^	3.9 ± 2.5		3.9 ± 3.1		0.805	
Main reason for infertility, n (%)					0.250	
Tubal factor	149 (44.3)		42 (37.8)			
Male factor	105 (31.3)		46 (41.4)			
Unexplained	58 (17.3)		15 (13.5)			
Combined	24 (7.1)		8 (7.2)			
Fertilization procedure, n (%)^b^					0.212	
IVF	182 (54.2)		50 (45.5)			
ICSI	130 (38.7)		53 (48.2)			
IVF and ICSI	24 (7.1)		7 (6.4)			
Basal FSH level, mIU/ml^a^	3.0 ± 0.9		3.1 ± 1.0		0.827	
Basal E_2_ level, pg/mL^a^	21.4 ± 12.4		20.4 ± 10.6		0.498	

^a^ Values are mean±SD

^b^ Total number of evaluable patients were 110 for Gonal-F^®^ Pen group.

**Table 2:** Efficacy outcomes (PP population).

	**Follitrope^TM^ PFS** **(N = 336)**	**Gonal-F^®^ Pen** **(N = 110)**	**p-value**
Total injected dose of r-FSH, IU^a^	1945.3 ± 635.7	2020.2 ± 562.7	0.271
Duration of treatment, days^a^	10.7 ± 1.6	11.1 ± 1.4	0.027
No. of follicles with diameter of ≥14 mm on hCG day ^a^	10.2 ± 4.0	10.1 ± 4.4	0.750
E_2_ concentration on hCG day, pg/mL ^a^	4460.0 ± 2564.1	4051.9 ± 2583.5	0.167
No. of oocytes retrieved^c^	14.9 ± 0.5	12.8 ± 0.9	0.057
No. of Metaphase II oocytes, n (%)^a,b^	85.3 ±14.0	85.0 ± 15.8	0.895
Fertilization rate (%)	70.6	74.6	0.079
No. of embryos transferred^a^	2.0 ± 0.3	2.0 ± 0.4	0.731
Embryo implantation rate (%)	39.8 (144/362)	35.5 (55/155)	0.376
Biochemical pregnancy rate (%)	4.3 (8/186)	10.1 (8/79)	0.110
Clinical pregnancy rate (%)	55.4 (103/186)	51.9 (41/79)	0.168
Ongoing pregnancy rate (%)	44.1 (82/186)	43.0 (34/79)	0.758

^a^ Values are mean±SD

^b^ ICSI patients only

^c^ Values are mean±SE

