# Supplementary material for: Efficacy and safety of recombinant human follicle-stimulating hormone in patients undergoing in vitro fertilization-embryo transfer
Source: Aging (Albany NY). 2020 Mar 25;12(6):4918–30. doi: 10.18632/aging.102919 (PMC7138541; doi:10.18632/aging.102919)
Supplement: Supplementary File [file aging-12-102919-s001..pdf]

## SUPPLEMENTARY MATERIAL

## Study Inclusion/Exclusion Criteria

**Inclusion criteria**

1. Women aged between 20 and 39 years;
2. A menstrual cycle of 25–35 days;
3. Normal baseline serum follicle-stimulating hormone (FSH), luteinizing hormone (LH), estradiol, and progesterone levels;
4. Indications for IVF-ET:
   - Tubal factors
   - Unexplained infertility
   - Male infertility
   - Combined factors
5. Voluntary to sign the Informed Consent Form (ICF)

**Exclusion criteria**

1. Presence of clinically significant major systemic disease or endocrine or metabolic disorders;
2. BMI >30 kg/m$^2$;
3. Presence of abnormalities of the uterus (uterine fibroids [equal to or greater than 2 cm in diameter or affecting the endometrium], endometrial polyp, intrauterine adhesion, uterine malformation), ovary (polycystic ovary, ovarian cyst), or appendix (hydrosalpinx) at the time of randomization;
4. A medical history of uterine/ovarian surgery;
5. A history of ovarian hyperstimulation syndrome (OHSS) in previous IVF cycles;
6. Poor response to previous gonadotropin therapy;
   * Presence of the following:
   ① previous poor ovarian response (less than 3 oocytes through previous conventional stimulation protocol)
   ② Abnormal ovarian reserve testing (i.e., <5 antral follicles)
7. Abnormal uterine bleeding of unknown cause;
8. At least one member of the couple will receive donor sperms or donor eggs, or preimplantation genetic diagnosis;
9. Positive for HIV or syphilis;
10. Presence of known serious mental disorders or inability to understand the purpose, methods, and other aspects of this clinical study and to follow the study procedures;
11. A habit of alcoholism, heavy smoking, and/or drug abuse;
12. A history of allergy to recombinant human follicle-stimulating hormone (r-hFSH) or any of its excipients;
13. Subjects who are found to participate in any other clinical study following enrollment in this study or who have participated in another clinical study 3 months prior to randomization in this study;
14. A medical history of ovarian, breast, uterine, or hypothalamic or pituitary tumors;
15. More than 3 previous consecutive failed IVF cycles;
16. Clomiphene or gonadotropin therapy within 1 month prior to randomization;
17. Patients with positive results in serum pregnancy test;
18. Failure to reach the standard (antral follicles size ≥10 mm) after 14 ± 2 days of downregulation
