# Supplementary material for: Efficacy and safety of recombinant human follicle-stimulating hormone in patients undergoing in vitro fertilization-embryo transfer
Source: Aging (Albany NY). 2020 Mar 25;12(6):4918–30. doi: 10.18632/aging.102919 (PMC7138541; doi:10.18632/aging.102919)
Supplement: Supplementary Table 1 [file aging-12-102919-s002..pdf]

SUPPLEMENTARY TABLE

Supplementary Table 1. Efficacy outcomes by age subgroup (PP population).

		Follitrope™ PFS (N = 336)	Gonal-F® Pen (N = 110)	*p*-value
Total number of oocytes retrieved[a]		15.4 ± 7.5	13.9 ± 6.4	-
20-30 yrs	N	198	73	
	Mean ± SD	16.3 ± 7.8	15.4 ± 6.8	1.0 (95% CI: [-1.1, 3.0])[b]
31-35 yrs	N	118	30	
	Mean ± SD	14.2 ± 6.8	11.7 ± 4.2	2.5 (95% CI: [0.5, 4.5])[b]
36-39 yrs	N	20	7	
	Mean ± SD	12.5 ± 7.0	7.4 ± 3.2	5.1 (95% CI: [−0.7, 10.8])[b]
Total injected dose of r-FSH, IU[a]		1945.3 ± 635.7	2020.2 ± 562.7	0.271
20-30 yrs	N	198	73	
	Mean ± SD	1772.5 ± 538.0	1869.9 ± 495.7	0.178
31-35 yrs	N	118	30	
	Mean ± SD	2122.8 ± 630.3	2227.5 ± 565.8	0.409
36-39 yrs	N	20	7	
	Mean ± SD	2610.0 ± 840.3	2700.0 ± 476.3	0.792
Duration of treatment, days[a]		10.7 ± 1.6	11.1 ± 1.4	0.027
20-30 yrs	N	198	73	
	Mean ± SD	10.7 ± 1.6	11.1 ± 1.5	0.086
31-35 yrs	N	118	30	
	Mean ± SD	10.6 ± 1.5	11.0 ± 1.1	0.081
36-39 yrs	N	20	7	
	Mean ± SD	11.1 ± 1.9	11.1 ± 1.7	0.958
Biochemical pregnancy rate (%)		4.3 (8/186)	10.1 (8/79)	0.110
20-30 yrs	N	98	46	
		5.1 (5/98)	4.3 (2/46)	0.464
31-35 yrs	N	74	27	
		2.7 (2/74)	14.8 (4/27)	0.042
36-39 yrs	N	14	6	
		7.1 (1/14)	33.3 (2/6)	0.202
Clinical pregnancy rate (%)		55.4 (103/186)	51.9 (41/79)	0.168
20-30 yrs	N	98	46	
		55.1 (54/98)	58.7 (27/46)	0.798
31-35 yrs	N	74	27	
		60.8 (45/74)	51.9 (14/27)	0.180
36-39 yrs	N	14	6	
		28.6 (4/14)	0 (0/6)	0.214
Ongoing pregnancy (%)		44.1 (82/186)	43.0 (34/79)	0.758
20-30 yrs	N	98	46	
		41.8 (41/98)	50.0 (23/46)	0.641
31-35 yrs	N	74	27	
		51.4 (38/74)	40.7 (11/27)	0.539
36-39 yrs	N	14	6	
		21.4 (3/14)	0 (0/6)	0.521

[a]Values are mean±SD; [b]Treatment differences and its 95% confidence interval instead of p-value.
